# Comparing the old and new generation SELDI-TOF MS: implications for serum protein profiling

**DOI:** 10.1186/1755-8794-1-4

**Published:** 2008-01-31

**Authors:** Marie-Christine W Gast, Judith YMN Engwegen, Jan HM Schellens, Jos H Beijnen

**Affiliations:** 1 Department of Pharmacy & Pharmacology, The Netherlands Cancer Institute/Slotervaart Hospital, Amsterdam, The Netherlands; 2 Department of Medical Oncology, The Netherlands Cancer Institute/Antoni van Leeuwenhoek Hospital, Amsterdam, The Netherlands; 3 Faculty of Science, Department of Pharmaceutical Sciences, Division of Biomedical Analysis, Utrecht University, Utrecht, The Netherlands

## Abstract

**Background:**

Although the PBS-IIc SELDI-TOF MS apparatus has been extensively used in the search for better biomarkers, issues have been raised concerning the semi-quantitative nature of the technique and its reproducibility. To overcome these limitations, a new SELDI-TOF MS instrument has been introduced: the PCS 4000 series. Changes in this apparatus compared to the older one are a.o. an increased dynamic range of the detector, an adjusted configuration of the detector sensitivity, a raster scan that ensures more complete desorption coverage and an improved detector attenuation mechanism. In the current study, we evaluated the performance of the old PBS-IIc and new PCS 4000 series generation SELDI-TOF MS apparatus.

**Methods:**

To this end, two different sample sets were profiled after which the same ProteinChip arrays were analysed successively by both instruments. Generated spectra were analysed by the associated software packages. The performance of both instruments was evaluated by assessment of the number of peaks detected in the two sample sets, the biomarker potential and reproducibility of generated peak clusters, and the number of peaks detected following serum fractionation.

**Results:**

We could not confirm the claimed improved performance of the new PCS 4000 instrument, as assessed by the number of peaks detected, the biomarker potential and the reproducibility. However, the PCS 4000 instrument did prove to be of superior performance in peak detection following profiling of serum fractions.

**Conclusion:**

As serum fractionation facilitates detection of low abundant proteins through reduction of the dynamic range of serum proteins, it is now increasingly applied in the search for new potential biomarkers. Hence, although the new PCS 4000 instrument did not differ from the old PBS-IIc apparatus in the analysis of crude serum, its superior performance after serum fractionation does hold promise for improved biomarker detection and identification.

## Background

The development of mass spectrometry (MS) for the analysis of complex protein mixtures has greatly enhanced the possibility of large-scale protein profiling studies. Protein profiling studies are generally performed using a top-down approach starting with a mixture of intact proteins and peptides. After sample pre-fractionation, e.g. by two-dimensional polyacrylamide gel electrophoresis (2D-PAGE), proteins are identified either by peptide mass fingerprinting using tryptic digestion and/or tandem MS. Mass spectrometry for protein profiling is particularly important for the low-molecular-weight fraction of the proteome, since the use of immunological assays is limited due to a lack of antibodies for these peptides. Up until recently, real high-throughput technologies for mass spectrometric protein profiling have been lacking. Two recent applications of matrix-assisted laser desorption ionisation – time of flight mass spectrometry (MALDI-TOF MS) combine sample pre-fractionation with MS, facilitating the analysis of many samples at the time. A magnetic beads-based assay using beads with different chromatographic affinities is available from Bruker Daltonics [1]. Alternatively, surface-enhanced laser desorption ionisation – time of flight mass spectrometry (SELDI-TOF MS; Bio-Rad Laboratories, Hercules, CA) can be used to profile biological matrices on arrays with different surface chemistries. A ProteinChip Interface is available for hybrid quadrupole – time of flight mass spectrometers (PCI-QqTOF; e.g. QSTAR, Applied Biosystems/MDS SCIEX, Foster City, CA, USA), permitting QqTOF analysis of ProteinChip arrays. Although QqTOF platforms have both tandem MS capability and superior mass accuracy, it suffers from decreased sensitivity and a limited data acquisition range (up to 4 kDa), compared to the SELDI-TOF MS platform (up to 200 kDa).

The SELDI-TOF MS technology has been extensively used for the assessment of tissue, serum and plasma to find diagnostic, prognostic or therapy-predictive biomarkers for diseases, especially cancer [2-8]. However, issues have been raised concerning the semi-quantitative nature of the technique and its reproducibility [9-11]. The first generation SELDI-TOF MS instruments (PBS-II and PBS-IIc) generate spectra with a fixed maximum signal, which is set to 100. Protein abundances exceeding this maximum saturate the detector and are cut off to 100, neglecting the excess abundance and leading to underestimated peak intensities from both the saturated peak and its following peak, as the detector remains saturated for some time [12]. Furthermore, settings for laser intensity and detector sensitivity are not easily optimised to generate unsaturated spectra for all the samples to be measured. To overcome these limitations a new SELDI-TOF MS instrument has been introduced: the PCS 4000 series. Changes in this apparatus compared to the older ones are: 1) the increased dynamic range of the detector, so that saturation is less likely to occur, 2) the special configuration for sensitivity in the high mass range for better detection of proteins > 100 kDa, 3) a so-called Synchronised Optical Laser Extraction, which scans each spot in a raster to ensure complete desorption coverage, 4) a detector attenuation mechanism, enabling signal reduction up to a specified mass and preventing saturation by matrix molecules. Furthermore, instead of using arbitrary units, peak intensities are scaled in μA, corresponding to the real electric current generated by the impact of ions onto the detector. Laser intensity settings are in nJ [13].

These improvements should lead to better reproducibility of peak intensities and detection of more peaks. Yet, the ultimate gain would be that this leads to more and better biomarker candidates. We chose to assess these claims by serum protein profiling of two different cohorts of cancer patients and matched controls on both the PBS-IIc and the PCS 4000 SELDI-TOF MS. The data generated on each platform were analysed by the associated software packages. Furthermore, the PBS-IIc generated data were analysed by the software package associated with the PCS 4000 apparatus, to assess the influence of the different software packages. The numbers of detected and significantly different peaks on both instruments were compared, as was the potential of each data set to yield a reliable classification of patients and controls. Furthermore, the reproducibilities of the instruments were compared. Lastly, we also profiled serum fractions and assessed the difference in number of peaks detected between the PBS-IIc and PCS 4000 instruments.

## Methods

### Chemicals

All used chemicals were obtained from Sigma, St. Louis, MO, USA, unless stated otherwise.

### Patient samples

The performances of both apparatus were assessed with two distinct sample sets. A first set of 45 sera from colorectal cancer (CRC) patients and 43 matched controls (CON) was prospectively collected between July 2003 and October 2005 (referred to as the CRC set). The second set consisted of 45 sera from breast cancer (BC) patients and 46 matched normal women (CON), collected between January 2003 and July 2005 (referred to as the BC set). Both sets were obtained at the Netherlands Cancer Institute, in Amsterdam, The Netherlands. Sample collection was performed with individuals' informed consent after approval by the institutional review boards.

### Serum fractionation

Serum samples from three normal women were fractionated in duplicate on QhyperD beads with a strong anion exchange moiety (Bio-Rad Labs), according to manufacturer's protocol. Sample fractionation was performed with a Biomek 3000 Laboratory Automation Workstation (Beckman Coulter Inc.). First, sera were denatured with 9 M urea/2% 3 [(3-cholamidepropyl)-dimethylammonio]-propane sulfonate (CHAPS). After binding of denatured serum to the beads, the flow-through was collected and bound proteins were subsequently eluted with buffers with pH from 9 to 3. Remaining proteins were finally eluted with an organic buffer.

### Protein profiling

For profiling of whole serum each sample was analysed according to previously developed protocols [4]. CRC samples and their matched controls were first denatured with 9 M urea/2% CHAPS/1% dithiotreitol. Then, each sample was applied in triplicate on CM10 arrays (weak cation exchange chromatography) with 20 mM sodium phosphate pH 5/0.1% TritonX-100 as a binding buffer and 20 mM sodium phosphate pH 5 as a wash buffer. BC samples and their matched controls were denatured in 9 M urea/2% CHAPS, after which each sample was applied in duplicate on IMAC30 arrays (immobilised metal affinity capture chromatography). Prior to sample application, IMAC30 arrays were charged twice with 50 μL 100 mM nickel sulphate (Braun, Emmenbrücke, Germany), followed by three rinses with deionised water. Phosphate Buffered Saline (PBS; 0.01 M) pH 7.4/0.5 M sodium chloride/0.1% TritonX-100 was applied as a binding buffer and PBS pH 7.4/0.5 M sodium chloride as a wash buffer. For both sample sets, a 50% sinapinic acid (SPA; Bio-Rad Labs) solution in 50% acetonitrile (ACN)/0.5% trifluoroacetic acid (TFA) was used as energy absorbing matrix.

Profiling of fractionated serum was performed on both CM10 chips and IMAC30 arrays. Binding and wash buffers were 100 mM sodium acetate pH 4 and 50 mM HEPES buffer for CM10. For IMAC30, 100 mM copper sulphate was used as charging solution, 100 mM sodium acetate as neutralizing buffer and 100 mM sodium phosphate pH 7/0.5 M sodium chloride as binding and wash buffer (all: Bio-Rad Labs). A solution of 50% SPA in 50% ACN/0.5% TFA was used as matrix.

During all profiling experiments arrays were assembled in 96-well format bioprocessors (Bio-Rad Labs), which were placed on a platform shaker at 350 rpm. Arrays were equilibrated twice with 200 μL of binding buffer, incubated with denatured sample or QHyperD serum fraction for 30 min and, after binding, washed twice with binding buffer, followed by two washes with wash buffer. Lastly, arrays were rinsed with deionised water. After air-drying, two times 1 μL of matrix was applied to the array spots.

SELDI-TOF MS analysis of all datasets was performed with both the PBS-IIc and the PCS 4000 ProteinChip Reader (Bio-Rad Labs). Data acquisition and processing were optimised for each sample set separately. Each spot was read twice, once with the PCS 4000 and once with the PBS-IIc instrument. Measurement settings for each apparatus and sample set are summarised in Table 1. M/z values were calibrated externally with All-in-One peptide standard (Bio-Rad Labs).

**Table 1:** Settings for protein profiling and data processing

	**CRC sample set**		**BC sample set**	
	**PBS-IIc**	**PCS 4000**	**PBS-IIc**	**PCS 4000**

**SELDI analysis parameters**				

Samples	CRC: n = 45 CON: n = 43		BC: n = 45 CON: n = 46	
Array type	CM10		IMAC30 Ni	
Binding conditions	20 mM NaAc pH 5		PBS pH 7.4/0.5 M NaCl	
Replicates	3		2	

**SELDI acquisition parameters**				

m/z range	0–200 kDa	0–200 kDa	0–200 kDa	0–200 kDa
Laser intensity	155	3500 nJ	155	3500 nJ
Detector sensitivity	6	n.a.	5	n.a
Deflector/detector attenuation	2000 Da	2000 Da	1000 Da	1000 Da
Laser shots kept	65	530	105	530
Not-assessable spectra	CRC: 2/135 CON: 4/129		BC: 4/90 CON: 0/92	
Cluster settings:				
First pass	S/N 5	Valley depth 5	S/N 5	Valley depth 5
Second pass	S/N 2	Valley depth 2	S/N 2	Valley depth 2
Cluster mass window	0.3%	0.3%	0.3%	0.3%
Present in	45%	45%	30%	30%

n.a.: not applicable

### Statistics and bioinformatics

To account for possible differences in data processing by the different software packages, data from the PBS-IIc were analysed with the ProteinChip Software, version 3.1 (Bio-Rad Labs) as well as with Ciphergen Express™ version 3.0.6. (Bio-Rad Labs). PCS 4000 data were only processed with the latter package. The PBS-IIc-generated spectra analysed by the ProteinChip Software and Ciphergen Express™ respectively will further be referred to as "data set 1a" and "data set 1b". The PCS 4000-generated spectra, analysed by Ciphergen Express™ will be referred to as "data set 2".

Spectra from the CRC and BC sets were analysed separately. Acquired spectra from each set were compiled and analysed as a whole. Both the ProteinChip and Ciphergen Express™ software spectra were baseline subtracted with the following settings: smooth before fitting baseline: 25 points, fitting width: 10 times expected peak width. Filtering was "on" using an average width of 0.2 times the expected peak width. The noise was calculated from 2000 or 1000 to 200,000 Da for the CRC and BC set respectively. Spectra were normalised to the total ion current in the same m/z range. For peak clustering with the ProteinChip Software, the Biomarker Wizard (BMW; Bio-Rad Labs) application was used. For clustering with the Ciphergen Express™ software (Bio-Rad Labs), identical clustering conditions were defined (see Table 1). In each set, peaks were auto-detected starting from 2000 Da.

For the CRC and BC set, peak intensities from the triplicate (CRC) and duplicate (BC) analyses were averaged and mean peak intensities between groups compared by the non-parametric Mann-Whitney U (MWU) test (p < 0.01 considered statistically significant). For the CRC set, the median CV in each sample set was calculated from the CV's of the triplicate analyses for all clustered peaks in each data set as well as for all common peaks present in each of the three data sets.

For the BC set, Spearman's rank correlation coefficient was calculated on peak intensities in each duplicate analysis for all three data sets. The majority of peaks (> 50%) detected spectrum wide in the three data sets are of relatively low average intensity (< 5), increasing the chance of finding potential biomarkers in the low intensity range. Hence, the reproducibility of peaks in the low intensity range is of special interest. However, correlation analyses are influenced by outliers (e.g. the few high intensity peaks detected), even when using non-parametric statistics. We therefore chose to assess the reproducibility in subsets of peaks, starting with inclusion of the 10%, 20%, 30%, *etc*. of peaks with lowest intensity, and ending with inclusion of all peaks detected. Spearman's rank correlation coefficient and corresponding p-values were subsequently plotted per subset of peaks.

Classification performance of the data sets obtained with both apparatus was assessed by building classification trees with the Biomarker Patterns Software (BPS; Bio-Rad Labs). Trees were generated with the gini method and the minimal cost tree was chosen in both the CRC and BC sample set. A ten-fold cross validation was used to estimate the sensitivity and specificity for each tree.

For the CM10 and IMAC30 serum fractionation sets, baseline correction and noise calculation was performed as described for the CRC and BC set. For each duplicate fraction, peaks were auto-detected by the ProteinChip software or Ciphergen Express™ by the settings described in Table 1. The number of peaks in each fraction was assessed, as well as the number of unique peaks across all fractions.

## Results

### Protein profiling CRC set

Six spectra did not contain a protein profile and were thus not assessable (Table 1). For data set 1a normalisation factors as estimated by the apparatus-associated software of the assessable spectra were 0.67 to 3.39 (log -0.18 to 0.53) and for data set 1b 0.62 to 2.4 (log -0.21 to 0.38). For data set 2 values ranged from 0.33 to 4.8 (log -0.49 to 0.68). Since the spectra with aberrant normalisation factors (>2 SD from mean of log normalisation factor) were mostly not from the same samples for the two apparatus, none were excluded, to ensure an equal comparison of both machines. This concerned 13 and 11 spectra from data set 1a and 1b, and 14 from data set 2.

Comparing CRC vs. CON, 32 clusters were generated for data set 1a (Table 2). In contrast, despite similar settings for processing and the same spectra, only 27 clusters were generated for data set 1b. With the PCS 4000 (data set 2) 48 clusters could be detected. Although the number of detected peaks was highest for data set 2, data set 1a yielded a similar number of significantly different peaks. Detailed peak cluster information can be found in Table 3. Overall, the significantly different peaks in all data sets were largely similar.

**Table 2:** Peak clustering results for the CRC and BC sample sets

**Number of peaks detected in:**	**CRC sample set**		**BC sample set**	
	
	**all**	p* < 0.01	**all**	p* < 0.01
Data set 1a	32	19	81	47
Also detected in data set 1b	31	13	30	22
Also detected in data set 2	31	15	43	28
Data set 1b	27	14	31	22
Also detected in data set 2	26	11	29	21
Data set 2	48	20	59	45

* MWU test

**Table 3:** Peak cluster information for the CRC and BC sample set

**CRC sample set clusters (Da)**			**BC sample set clusters (Da)**		
**Data set 1a**	**Data set 1b**	**Data set 2**	**Data set 1a**	**Data set 1b**	**Data set 2**

2746*	2745*	2746*	2027		2028
		3163	2146*		2146*
		3406*	2154*		
3979*		3978*	2235*		
4160	4162	4159	2277*		
4179*	4181*	4182*	2647		
4290*		4287*	2675*		
		4303	2731*		
		4446*	2747		
4481	4474	4480	2760		2760*
4605		4607	2775		
		4961	2794		
5723*	5724*	5719*	2888*		
5913*		5915*	2960*		2961*
6443	6443	6442	2968*		
6459*	6460*	6458*	3091		
6641	6640	6643	3107		
6655*	6654*	6659*	3151*		
		6687	3168*	3165*	3164*
6846			3282*	3281*	3281*
	6860*	6865*	3296*		
7778	7779	7778	3431		
7982*	7982*	7990*	3451		
8079*		8074*	3689*		3683*
		8159	3781*		
		8889	3824		
8968*	8962*	8962*	3891*		3891*
9186	9187		3898		
		9210	3916*		
9307*	9307*	9315	3965*	3963*	3962*
		9360	3980*	3980*	3979*
		9409	3995*	3997*	3994*
		9593	4078		
		10072*	4137		4138
		12889	4155		
13779*	13778*	13796	4204*		
14077*	14077*	14053	4218*	4218*	4218*
15121	15121	15168	4292*	4292*	4289*
15930*	15919*	15982*	4308*	4308*	4304*
16105*	16105*	16139*	4334*		
		16334*	4449*	4447*	4444*
		18617*	4464*	4463*	4458*
23426*	23426	23486	4484*	4484*	4482*
28098*	28093	28216	4497*		
32308*	32308*	32394*	4513*		
		39727	4653	4652	4650
51034	51006	51116	4669		
56408	56337	56685	4691		
67003	67003	67239	4798		
79100	79040	80075	5076		5078
			5090		
			5274*		
			5348*	5348*	5348*
			5363*	5364*	5360*
			5554*		5549*
			5815		5810
			5916*	5917*	5915*
			5932*	5932*	5929*
			6100*	6097*	
			6122*	6122*	6121*
			6142*		6136*
			6667		6676
			6848		
			6965*	6972*	6966*
			6990*		
			7482		
			7778	7778	7775
			7939*		
			7985		7982
			8155	8155	8150
			8948*	8955*	8946*
			9161*		9151*
			9302	9303	9299
					9526
					11096*
					11747*
			13925*	13925*	13919*
					14124*
					22284*
					28221
					30502*
			33475*	33583*	33490*
					40059*
			43108	43015	43098*
					50704*
			60776	60804	60889*
			66702*	66711*	67142*
			79724	79393	80323*
					89750*
					91037
					93689*
					104178*
			109494		110344*
			133447	133435	
					136611*
				149723	149579*
					177011

* MWU test; p < 0.01

Classification trees were built with all clustered peaks in each data set and with the subset of the 25 clusters that were detected in all three data sets (Table 4). The best tree was generated with data set 2, with m/z 4446 as single classifier and sensitivity and specificity ≥ 80%. Since this peak was not detected in data set 1a and 1b, other peaks were used as classifiers in these sets, respectively m/z 15930 and 32308. The classification trees constructed on the subset of 25 common clusters in data sets 1a and 1b applied the same cluster (m/z 32308). The best classifier of data set 2 made use of apparently the same cluster (m/z 32394), and had a better performance as single classifier in this set than in set 1a, but a similar performance as in set 1b.

**Table 4:** Characteristics of the classification trees constructed on the CRC sample set

**Tree characteristics:**							Tree performance*:	
**Data set**	**Clusters**	**(#)**	**Node 1**		**Node 2**		**Sens (%)**	**Spec (%)**

1a	All	(32)	m/z 15930	≤ 35.576	m/z 51034	≤ 1.372	68.8	62.8
	Common	(25)	m/z 15930	≤ 35.576	m/z 51034	≤ 1.372	68.8	62.8
1b	All	(27)	m/z 32308	≤ 0.676	-	-	75.6	73.8
	Common	(25)	m/z 32308	≤ 0.676	-	-	75.6	73.8
2	All	(48)	m/z 4446	≤ 1.136	-	-	82.2	90.5
	Common	(25)	m/z 32394	≤ 0.149	-	-	73.3	81.0

* tree performance as determined by 10-fold cross validation, sens: sensitivity, spec: specificity

### Protein profiling BC set

Following array reading with both the PCS 4000 and PBS-IIc apparatus, two spectra did not contain a protein profile. Along with their duplicate reading, these spectra were excluded from further analyses (Table 1). The normalisation factors for data set 1a were 0.52 to 2.15 (log -0.29 to 0.33). Using Ciphergen Express™ software, normalisation factors of all spectra ranged from 0.51 to 2.25 (log -0.29 to 0.35) for the PBS-IIc generated spectra and from 0.44 to 2.69 (log -0.36 to 0.43) for the PCS 4000 generated spectra. In total, 9 and 8 spectra from data set 1a and 1b respectively and 10 spectra from data set 2 had an aberrant normalisation factor (>2 SD from mean of log normalisation factor). As the majority of these spectra were from different samples for the 3 datasets, none were excluded, to ensure equal comparison of both apparatus. In data set 1a and 1b respectively, a total of 81 and 31 clusters were detected. The ProteinChip software detected 51 clusters that were not detected by Ciphergen Express™ in the same dataset. Except for one cluster (>100 kDa), these unique clusters were all < 10 kDa in mass and < 4 in intensity. In the data set 2, a total of 59 peak clusters was detected. Fifteen of these clusters (all > 9 kDa) were not detected in either data set 1a or 1b. Tables 2 and 3, respectively, provide an overview of peak clustering results and detailed peak cluster information.

Classification trees were generated on all peaks detected in data set 1a, 1b or 2, and on the subset of peaks detected across all three data sets (Table 5). All optimum decision trees constructed on data set 1a and 1b, using either all peaks detected or only the common peaks, applied m/z 3964 as single classifier, with data set 1b yielding the best performance of ~80%. The trees constructed on data set 2 made use of different clusters, either considering all peaks detected (m/z 9151 and m/z 5360) or the common peaks detected (m/z 3979 and m/z 4218). However, the tree constructed on data set 1b generally had the best performance.

**Table 5:** Characteristics of the classification trees constructed on the BC sample set

**Tree characteristics:**							Tree performance*:	
**Data set**	**Clusters**	**(#)**	**Node 1**		**Node 2**		**Sens (%)**	**Spec (%)**

1a	All	(81)	m/z 3964	≤ 4.010	-	-	74.4	73.9
	Common	(28)	m/z 3964	≤ 4.010	-	-	74.4	73.9
1b	All	(31)	m/z 3964	≤ 3.855	-	-	83.7	78.3
	Common	(28)	m/z 3964	≤ 3.855	-	-	83.7	78.3
2	All	(59)	m/z 9151	≤ 1.614	m/z 5360	≤ 17.856	72.1	78.3
	Common	(28)	m/z 3979	≤ 32.163	m/z 4218	≤ 4.648	62.8	71.7

* tree performance as determined by 10-fold cross validation, sens: sensitivity, spec: specificity

### Reproducibility CRC set

For each data set on each apparatus the inter-chip reproducibility was assessed by calculating the median CV across all samples from replicate peak intensities of all clustered peaks and of the subset of 25 common peaks in the three data sets. The median CV of all peaks and all common peaks was lowest for data set 2 and highest for data set 1a (Table 6). Considering all peaks, the CV was significantly different for the data sets (Kruskall Wallis test; p = 0.012), but not when considering only the common peaks to each data set (Kruskall Wallis test; p = 0.3).

**Table 6:** Reproducibility of the CRC data sets

**Peak clusters:**	**Median CV of:**		
	
	**Data set 1a**	**Data set 1b**	**Data set 2**
All peaks	28.30%	22.61%	20.62%
Common peaks	25.48%	23.06%	21.71%
All peaks (CON)	28.52%	21.76%	18.50%
All peaks (CRC)	27.68%	23.38%	23.33%

### Reproducibility BC set

**Figure 1:**
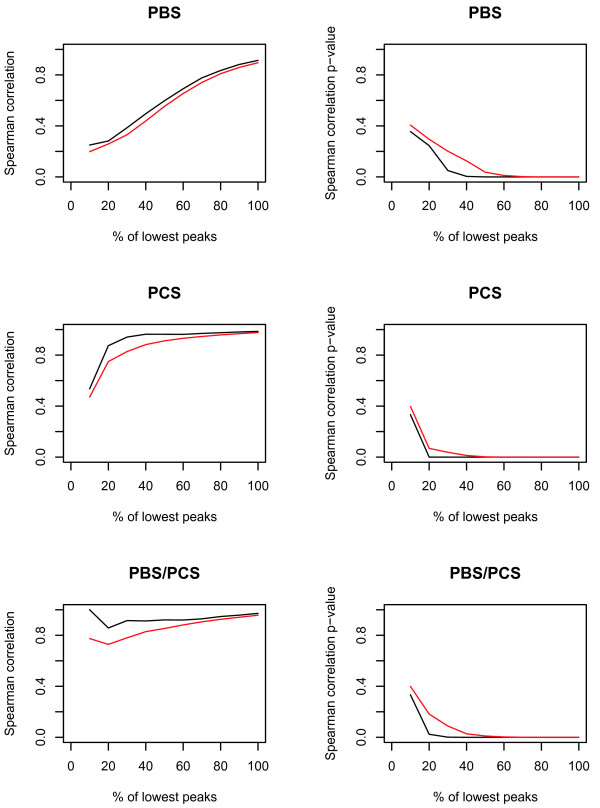
**Plots of Spearman's rank correlation coefficient and p-values for all peaks detected in the BC data sets**. Depicted are the mean (red) and median (black) values of all peaks detected in the three data sets of the BC sample set. PBS: data set 1a (PBS-IIc generated data, analysed by ProteinChip software), PCS: data set 2 (PCS 4000 generated data, analysed by Ciphergen Express™), PBS/PCS: data set 1b (PBS-IIc generated data, analysed by Ciphergen Express™).

**Figure 2:**
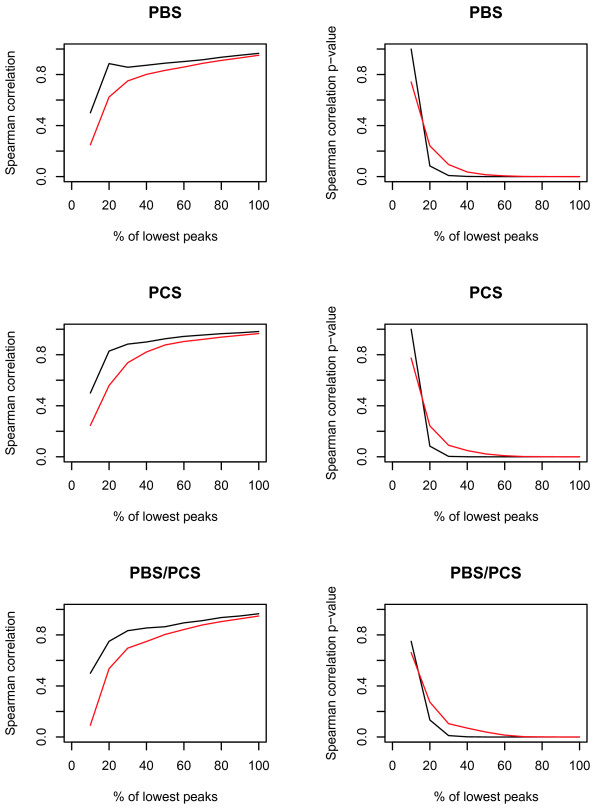
**Plots of Spearman's rank correlation coefficient and p-values for common peaks detected in the BC data sets**. Depicted are the mean (red) and median (black) values of common peaks detected across all three data sets of the BC sample set. PBS: data set 1a (PBS-IIc generated data, analysed by ProteinChip software), PCS: data set 2 (PCS 4000 generated data, analysed by Ciphergen Express™), PBS/PCS: data set 1b (PBS-IIc generated data, analysed by Ciphergen Express™).

For the BC sample set, Spearman's rank correlation coefficient was calculated on peak intensities in each duplicate analysis for successive subsets of peaks including the 10%, 20%, 30% to 100% of peaks with lowest intensity, for all three data sets. As depicted in Figure 1, a correlation coefficient > 0.8 was only reached after inclusion of 80% of lowest peaks in data set 1a, while in the other two data sets, this coefficient was already reached at inclusion of < 20% of lowest peaks. Similar results were obtained when considering the significance of correlation (Figure 1). However, when considering only the common peaks detected across all three data sets, results obtained were highly similar for the three data sets (Figure 2).

### Serum fractionation

**Figure 3:**
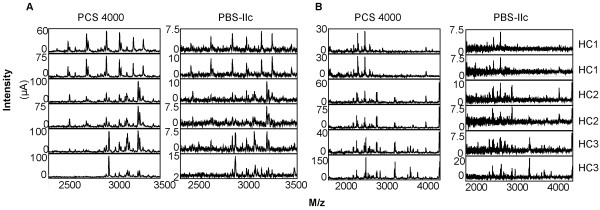
**Spectra of serum fractions analysed on CM10 arrays and measured on the PBS-IIc and PCS 4000 instrument**. A: flow through/pH 9 fractions, B: pH 7 fractions.

The numbers of clusters detected on CM10 and IMAC arrays for each sample in each acquired fraction are summarised in Table 7. Some of the clusters are occurring in several fractions. Ignoring these overlapping clusters, on average twice as many peaks were detected in the PCS 4000 generated spectra compared to the PBS-IIc generated spectra (analysed either by the ProteinChip software or Ciphergen Express™). This is also illustrated in Figure 3.

**Table 7:** Peak clustering results for the serum fractions profiled on CM10 and IMAC30 arrays

**Data set:**	**Serum sample 1**						**Serum sample 2**						**Serum sample 3**					
	
	**CM10**			**IMAC30**			**CM10**			**IMAC30**			**CM10**			**IMAC30**		
	
	**1a**	**1b**	**2**	**1a**	**1b**	**2**	**1a**	**1b**	**2**	**1a**	**1b**	**2**	**1a**	**1b**	**2**	**1a**	**1b**	**2**
FT+pH9	43	24	60	23	22	52	59	23	57	28	22	43	29	19	47	24	15	47
pH7	16	6	45	9	9	30	28	9	46	9	7	24	9	5	51	7	7	26
pH5	42	19	51	15	15	32	37	16	49	24	16	29	21	15	56	17	11	28
pH4	22	22	46	24	22	53	19	17	50	34	24	54	23	19	54	23	21	46
pH3	20	20	46	16	14	42	10	8	48	16	13	36	19	16	58	18	13	38
Organic	22	17	58	31	30	40	17	12	61	22	15	36	24	20	53	19	20	42
Total	165	108	306	118	112	249	170	85	311	133	97	222	125	94	319	108	87	227
Unique	103	78	167	85	82	158	106	61	162	82	72	135	82	71	163	67	53	128

## Discussion

Although the PBS-IIc SELDI-TOF MS apparatus has been extensively used in the search for better biomarkers, issues have been raised concerning the semi-quantitative nature of the technique and its reproducibility. To overcome these limitations, a new SELDI-TOF MS instrument has been introduced: the PCS 4000 series. In the current study, we compared the performances of the old PBS-IIc and new PCS 4000 series generation SELDI-TOF MS apparatus, by analysis of two sample sets.

### Peak detection

For the CRC sample set, most peaks were detected with the new PCS 4000 series using the Ciphergen Express™ software, indicating a better sensitivity and less detector saturation of this apparatus. The latter allows for the application of increased laser intensities, after which proteins will desorb more comprehensively, resulting in detection of more peaks. However, for the BC sample set, most peaks were detected with the PBS-IIc instrument using the ProteinChip software, indicating the opposite. Interestingly, in both sample sets, fewer peaks were detected by Ciphergen Express™ than by the ProteinChip software in the spectra generated with the PBS-IIc, despite the fact that both software packages use the same algorithm with similar settings to generate peak clusters. Apparently, the spectrum processing algorithms underlying the visible settings are different for both software packages.

In the BC set, all peaks detected in the PBS-IIc generated spectra by the ProteinChip software, but missed by Ciphergen Express™ were < 4 in intensity. As peaks are detected by means of their signal-to-noise ratio, detection of these low intensity peaks becomes critical when either the noise increases or the signal decreases due to over-estimation of the baseline. Conceivably, the algorithm for noise and/or baseline estimation between both software packages has been changed. Due to the detector attenuation of the PCS 4000 instrument, matrix blanking has improved compared to the PBS-IIc. Hence, less chemical noise is expected when measuring with the PCS 4000 instrument, to which the algorithm applied in noise calculation might have been adapted. As such, for spectra generated with the PBS-IIc (in which relatively more chemical noise is present), the Ciphergen Express™ software will estimate the noise too high or the signal too low, the latter being the consequence of the baseline being estimated too high. Either way results in fewer detected peaks.

The difference between peaks detected by either software package in the PBS-IIc generated spectra was more pronounced in the BC set than in the CRC set. These two datasets differed in their deflector/detector attenuation settings (CRC: 2000 Da, BC: 1000 Da), but in both sets, the noise was calculated between 2 and 200 kDa. However, as matrix peaks are generally observed up to 2000 Da, their contribution to the noise will most likely increase with decreasing deflector settings. Hence, the difference in deflector settings could have caused higher noise estimation in the BC set compared to the CRC set. Combined with the probable noise overestimation by Ciphergen Express™ in PBS-IIc generated spectra, and the fact that relative to the CRC data sets, the BC data sets contained more low intensity peaks (30 and 70%, respectively), which were mainly present in the <10 kDa range, this might explain the more pronounced difference in number of peaks detected in the PBS-IIc generated BC dataset by both software packages.

The difference in deflector/detector attenuation settings might also explain why, contrary to the CRC set, in the BC set more peaks were detected by the ProteinChip software in the PBS-IIc spectra than by Ciphergen Express™ in the PCS 4000 spectra. Compared to the ProteinChip software, the noise calculation algorithm in Ciphergen Express™ apparently is more sensitive to the noise in the low molecular weight range. Due to the difference in detector attenuation settings, this low molecular weight range will contain a higher signal in the BC spectra than in the CRC spectra. Consequently, the noise is estimated higher and less peaks are detected. This hypothesis is supported by the observation that all peaks detected in the PBS-IIc spectra, but not in the PCS 4000 spectra were < 3 in intensity.

One of the alleged improvements of the PCS 4000 compared to its PBS-IIc predecessor is its special configuration for sensitivity in the high mass range that allows detection of proteins above 100 kDa. Indeed, in the BC set, four > 100 kDa peaks were detected exclusively in the PCS 4000 generated spectra, compared to two peaks in the PBS-IIc generated spectra. Moreover, all peaks that were detected exclusively in the PCS 4000 spectra by Ciphergen Express™ were above 10 kDa. However, in none of the CRC data sets any proteins > 100 kDa were detected, indicating no better sensitivity for proteins in the higher mass range for the PCS 4000 series. Most peaks detected only in data set 2 were in the 2–10 kDa range. The differences in detection of high molecular weight peaks could, however, be caused by the different array types used for the analyses of both sample sets.

### Classification

As the ultimate gain of the improved performance of the PCS 4000 instrument would be detection of more and better biomarker candidates, we also assessed the classification potential of the data sets generated by both machines. For the CRC set, the improved performance of the new instrument was indeed reflected in the classifiers constructed, as the best classification was obtained with the data set generated by the PCS 4000 instrument, using the total number of peaks detected. When using the subset of peaks detected in all three datasets, the performance of the classifier build on dataset 1b and 2 was similar. For the BC data set, results were less unambiguous. While for data set 1a and 1b only one classifier was applied in the different optimum decision trees constructed, best performance was achieved in data set 1b. Apparently, the different spectrum processing algorithms underlying both software packages also contribute to the alleged improved performance of the PCS 4000 instrument. However, application of both the PCS 4000 and Ciphergen Express™ yielded no better classifiers. Hence, for the BC set, the superior performance of the PCS 4000 instrument in providing better biomarker candidates could not be confirmed. It can, however, not be precluded that our data sets do not contain any real biomarkers.

### Reproducibility

For the CRC set, the reproducibility of peak intensities was largely similar across data sets, although a non-significant trend could be seen to a lower CV for data set 2 compared to 1a and 1b. Thus, the spot scanning in a raster and the less detector saturation with the PCS 4000 series does not seem to result in a significant better reproducibility. The fact that significant differences in CV were seen when all peaks were considered indicates that the surplus of peaks detected in data set 2 consists of more robust peaks than the ones also detected in the other data sets, causing the median CV to drop. Reproducibility of the PCS 4000 instrument as measured by the CV has been stated to be < 20% using an external standard [13]. It is not known to us in which m/z range this reproducibility was obtained and whether this was with manual or robotic sample handling. However, our observed median CV is well in concordance with this value, especially taking the manual sample handling into account.

Reproducibility in the BC data sets was assessed by calculation of Spearman's rank correlation coefficient on duplicate intensities of the 10 to 100% peaks with lowest intensity. When all peaks detected were included in this calculation, usage of the PCS 4000 and Ciphergen Express™ software package led to a better performance, as statistically significantly (p < 0.05) good correlations (R > 0.8) were already achieved upon inclusion of only 20% of lowest peaks, compared to the 80% of lowest peaks necessary to achieve comparable results in the PBS-IIc generated data set. However, when correcting for the excess of low intensity peaks detected in data set 1a relative to data set 2 by considering only the peaks detected across all three data sets, results obtained were highly similar for the three data sets. Thus, the improved features of the PCS 4000 instrument relative to the PBS-IIc apparatus do not lead to an improved reproducibility, as already observed in the CRC data sets.

### Serum fractionation

Analysis of the PBS-IIc generated spectra by Ciphergen Express™ generally yielded the lowest number of peaks detected. Hence, the performance of the PCS 4000 in serum fractionation is indeed superior compared to the PBS-IIc instrument, reflecting the improved spot coverage and increased detector sensitivity. These observations are highly similar to the results obtained following peak detection in the three CRC data sets.

Although deflector/detector attenuation settings were different for the fractionation spectra on IMAC and CM10 chips, peak clustering results were highly similar for the two array types used, contrary to the results obtained in the CRC and BC sample sets. This could be due to the fact that these spectra have a higher noise level than spectra from crude serum (data not shown), limiting the influence of the different noise estimation between both software packages. Moreover, the number of peaks < 10 kDa is similar in the fractionation spectra from the IMAC and CM10 chips, contrary to the spectra from the CRC and BC set, which could also cause less influence of the noise estimation on peak detection.

## Conclusion

In conclusion, regarding the number of peaks detected, the biomarker potential and the reproducibility of the two sample sets investigated by both the old (PBS-IIc) and new (PCS 4000) generation SELDI-TOF MS apparatus, we could not confirm the alleged improved performance of the PCS 4000 instrument over the PBS-IIc apparatus. However, the PCS 4000 instrument did prove to be of superior performance in peak detection following profiling of serum fractions. Until now, the majority of studies in which SELDI-TOF MS was applied in crude serum protein profiling for biomarker discovery generally reported high abundant, non-disease-specific proteins as potential biomarkers. However, the large dynamic range of crude serum hampers detection of the allegedly high-informative low abundant serum proteins. As serum fractionation facilitates detection of low abundant proteins through reduction of this dynamic range, it is increasingly applied in the search for new potential biomarkers. Hence, although the new PCS 4000 instrument did not differ from the old PBS-IIc apparatus in the analysis of crude serum, its superior performance of fractionated serum samples does hold promise for improved biomarker detection and identification.

## Competing interests

The author(s) declare that they have no competing interests.

## Authors' contributions

MCWG and JYMNE participated in the design of the study, performed the laboratory work and the statistical analyses, and drafted the manuscript. JHMS and JHB conceived of the study, and helped to draft the manuscript. All authors read and approved the final manuscript.

## Pre-publication history

The pre-publication history for this paper can be accessed here:


